# Impact of Type 2 Diabetes Mellitus and Chronic Kidney Disease on Bone Mineral Density in Elderly Patients With Fragility Hip Fracture: A Cross‐Sectional Study

**DOI:** 10.1002/agm2.70035

**Published:** 2025-08-13

**Authors:** Debajyoti Roy, Jiashen Cai, Chee Yong Ng, Sreekanth Koduri, Chang Yin Chionh, Rehena Sultana, Wenxiang Yeon

**Affiliations:** ^1^ Department of Renal Medicine Changi General Hospital Singapore; ^2^ Centre for Quantitative Medicine Duke‐NUS Medical School Singapore

**Keywords:** bone mineral density, chronic kidney disease, diabetes mellitus, glycosylated hemoglobin

## Abstract

**Objectives:**

Diabetes mellitus (DM) and chronic kidney disease (CKD) are prevalent conditions in the elderly population, increasing the risk of bone fractures due to their adverse effects on bone quality. This study aimed to assess their impact on bone health and propose interventions to mitigate fracture risk in this demographic.

**Methods:**

A cross‐sectional study involving 571 elderly patients (aged ≥ 65 years) with fragility hip fractures was conducted at a tertiary care hospital between June 2014 and June 2016. Patients were categorized into four groups based on DM and CKD presence. Bone mineral density (BMD) at the femoral neck was measured using dual‐energy X‐ray absorptiometry (DXA) scan. Statistical analysis included ANOVA with Bonferroni correction.

**Results:**

The mean age was 79.5 ± 7.3 years, with females comprising 70.6%. Group 2 (No CKD with diabetes) exhibited higher *T*‐scores than Group 1 (No CKD or Diabetes). Patients with DM had higher *T*‐scores, with an increase of 0.2 compared to those without DM and CKD. Surprisingly, CKD and DM coexistence (Group 4) did not worsen *T*‐scores. Higher HbA1c levels were positively associated with a higher *T*‐score, but this was lost in concurrent CKD.

**Conclusions:**

Patients with DM had higher *T*‐scores; the combination of CKD and DM did not worsen *T*‐scores. However, the positive association between higher HbA1c and higher *T*‐score was nullified in concurrent CKD. These findings emphasize the need for tailored interventions to mitigate fracture risk in elderly populations with DM and CKD.

## Graphical abstract

Diabetes mellitus (DM) and chronic kidney disease (CKD) impact bone health in the elderly. A study of 571 patients revealed higher BMD in DM, with HbA1c positively linked to BMD. CKD nullified this association without worsening *T*‐scores. Tailored interventions—BMD monitoring, glycemic control, and fracture prevention—are essential for risk mitigation.

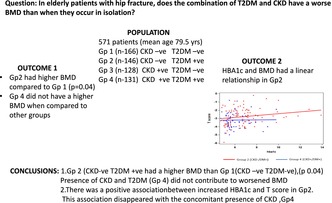

## 1 Introduction

Diabetes mellitus (DM) and chronic kidney disease (CKD) are common medical conditions among the elderly and have been shown to increase the risk of bone fractures due to their negative impact on bone quality. Recent data demonstrate the association of bone mineral density (BMD) measured by using dual‐energy X‐ray absorptiometry (DXA) scan with fragility fractures in patients with CKD [1].

The effect of type 1 diabetes mellitus (T1DM) and type 2 diabetes mellitus (T2DM) on BMD appears to be discordant [2]. Several studies have found a positive association between HbA1c levels and BMD in T2DM, independent of other factors such as age, sex, body mass index, and diabetes chronicity. The exact mechanisms underlying this relationship are not yet fully understood, but it has been suggested that hyperglycemia may affect bone cells and bone metabolism, leading to decreased bone formation and increased bone resorption [3, 4, 5]. In contrast, T1DM is associated with a lower BMD and increased fracture risk. Poorer glycemic control was associated with lower hip BMD in T1DM [6].

When DM and CKD occur in combination, the negative impact on BMD may be greater due to the complex interplay between the two conditions. Considering that a substantial number of patients in renal or geriatric clinics are affected by both, it is important for healthcare providers to be aware of the potential impact of DM and CKD on bone health and to implement appropriate interventions to reduce the risk of bone fractures in this population.

In this study, we investigate potential differences in the impact of T2DM and CKD on BMD in a cohort of elderly patients with fragility hip fracture when these conditions occur alone versus when they occur in combination. We also investigate the relationship of worsening HbA1c to BMD in the groups with CKD and T2DM versus T2DM alone.

## 2 Methods

### 2.1 Study Population

A cross‐sectional study was undertaken, encompassing 571 elderly patients aged ≥ 65 years, who experienced hip fractures and were admitted to Changi General Hospital between June 2014 and June 2016. The study received ethical approval from the Singhealth Institutional Review Board (Approval No: 2017/2962), with informed consent waived by the board. In a previous study utilizing the same dataset, we reported on the outcomes of hip fractures in the elderly [7].

Inclusion criteria were men and women over the age of 65 with hip fractures admitted to the hospital. At Changi General Hospital, the management of elderly patients with hip fractures falls under the Value Care Program, which emphasizes standardized evaluation and treatment protocols. This program aims to facilitate early surgical intervention and promotes a multidisciplinary approach to enhance patient outcomes and minimize complications. Surgical treatment is typically performed within 24–48 h of admission, and early ambulation is actively encouraged.

Exclusion criteria were an absence of serum creatinine measurements within 3 months prior to admission, acute kidney injury, history of malignancy, chronic liver disease, and absence of *T*‐score. The study population was stratified into four groups: Group 1—No CKD or diabetes (*n* = 166); Group 2—No CKD with diabetes present (*n* = 146); Group 3—CKD present with no diabetes (*n* = 128); Group 4—Both CKD and diabetes present (*n* = 131). All patients aged 65 years and above with hip fractures were included as part of the hospital's value care program. Data on patients admitted with hip fractures between June 1, 2014, and June 1, 2016, were extracted from electronic health records. Follow‐up extended from the index date to June 1, 2018, or until death.

### 2.2 Laboratory Values and BMD

The estimated glomerular filtration rate (eGFR) was calculated using the 2021 CKD‐EPI equation [8]. CKD was defined according to the KDIGO 2021 Clinical Practice Guidelines, including participants with CKD G3–5, corresponding to an eGFR ≤ 60 mL/min/1.73 m^2^ [9]. Biochemical values included 25(OH) vitamin D, serum albumin, serum calcium, and phosphate. BMD was measured using DXA at the lumbar spine (L1–L4) and femoral neck (Hologic QDR Discovery Wi, United States). We report *T*‐scores only at the femoral neck, as all patients in the cohort sustained hip fractures. Since this is a retrospective study, *T*‐scores at the lumbar spine were not available. The normative values for assessing BMD are derived from the local Singapore population, which is predominantly Chinese. These values are proprietary and specific to the Hologic densitometer. Studies in the local female population have indicated that BMD is 6%–8% lower at the femoral neck and 3%–8% lower at the AP spine compared to age‐matched Americans [10]. Similarly, an Asian male BMD reference database shows values that are 10% lower at the spine and 5% lower at the neck of femur compared to the Caucasian reference database [11]. Low BMD was defined as < 2.5 based on the WHO criteria for osteoporosis [12]. None of the patients were on anti‐osteoporosis therapy prior to sustaining a hip fracture. Standard anti‐osteoporotic treatment was initiated in the postoperative period, in accordance with current clinical guidelines.

### 2.3 Outcomes

We examined the impact of CKD and T2DM on BMD when these conditions were either present alone or in combination. Additionally, we studied the association of worsening HbA1c with BMD in the specified patient groups.

### 2.4 Statistical Analysis

Patients were stratified into four groups: Group 1‐No CKD or diabetes, Group 2‐No CKD with diabetes present, Group 3‐CKD present with no diabetes, Group 4‐both CKD and diabetes present. Continuous variables were summarized as mean ± standard deviation (SD) or median (interquartile range) and categorical variables as proportions. *p*‐values represent differences in characteristics based on ANOVA for continuous variables and Chi‐square test for categorical variables. A *p*‐value < 0.05 was considered significant. All statistical analyses were performed using SAS v9.4 (SAS Institute Inc., NC, USA).

Statistical significance for *p‐*values in the case of multiple comparisons was pre‐specified as 0.05/*n* using Bonferroni correction.

## 3 Results

### 3.1 Baseline Characteristics

A total of 571 elderly patients with hip fractures were included in the study and divided into four groups based on the presence or absence of CKD and DM. The groups were defined as follows:

Group 1: CKD−/DM− (*n* = 166).

Group 2: CKD−/DM+ (*n* = 146).

Group 3: CKD+/DM− (*n* = 128).

Group 4: CKD+/DM+ (*n* = 131).

The baseline characteristics of patients stratified by CKD and DM status are summarized in Table 1. The mean age of the study population was 79.5 ± 7.3 years, with 70.6% being female.

**TABLE 1:** Baseline Characteristics, by CKD and DM status.

Variables	CKD−/DM− *n =* 166	CKD−/DM+ *n =* 146	CKD+/DM− *n =* 128	CKD+/DM+ *n =* 131	Total *n =* 571	*p*
Demographics						
Age (years), mean (SD)	77.3 (6.88)	77.9 (7.29)	82.6 (6.42)	81.2 (7.16)	79.5 (7.28)	< 0.001
Sex, *n* (%)						0.556
Female	115 (69.3)	100 (68.5)	89 (69.5)	99 (75.6)	403 (70.6)	
Male	51 (30.7)	46 (31.5)	39 (30.5)	32 (24.4)	168 (29.4)	
BMI (kg/m^2^), mean (SD)	24.9 (1.71)	24.9 (1.61)	24.7 (1.43)	24.9 (1.69)	24.8 (1.61)	0.673
Comorbidities						
Diabetes, *n* (%)	0	146 (100)	0	131 (100)	277 (48.5)	< 0.001
Hypertension, *n* (%)	75 (45.2)	23 (15.8)	55 (43.0)	25 (19.1)	178 (31.2)	< 0.001
Hyperlipidemia, *n* (%)	107 (64.8)	37 (25.3)	72 (56.3)	37 (28.2)	253 (44.4)	< 0.001
Cerebrovascular accident, *n* (%)	14 (8.4)	6 (4.1)	10 (7.8)	12 (9.2)	42 (7.4)	0.361
Ischemic heart disease, *n* (%)	26 (15.7)	16 (11.0)	23 (18.0)	17 (13.0)	82 (14.4)	0.367
Congestive cardiac failure, *n* (%)	0	1 (0.7)	3 (2.3)	1 (0.8)	5 (0.9)	0.193
Paroxysmal atrial fibrillation, *n* (%)	1 (0.6)	0	1 (0.8)	3 (2.3)	5 (0.9)	0.215
Chronic obstructive pulmonary disease, *n* (%)	5 (3.0)	3 (2.1)	4 (3.1)	3 (2.3)	15 (2.6)	0.926
Peripheral vascular disease, *n* (%)	1 (0.6)	3 (2.1)	2 (1.6)	1 (0.8)	7 (1.2)	0.634
Malignancy, *n* (%)	14 (8.4)	3 (2.1)	9 (7.0)	9 (6.9)	35 (6.1)	0.109
Biochemical						
HbA1c (%), mean (SD)	5.5 (0.29)	6.9 (1.61)	5.4 (0.24)	6.8 (1.61)	6.5 (1.53)	< 0.001
eGFR, mL/min/1.73 m^2^, mean (SD)	60.0 (0.19)	60.0 (0.42)	44.5 (11.73)	43.0 (11.98)	52.7 (11.37)	< 0.001
Hemoglobin, g/dL, mean (SD)	12.4 (1.42)	13.1 (1.52)	12.2 (1.97)	11.7 (1.60)	12.4 (1.69)	< 0.001
Serum calcium, mmol/L, mean (SD)	2.2 (0.11)	2.3 (0.13)	2.3 (0.15)	2.2 (0.25)	2.2 (0.16)	0.446
Serum phosphate, mmol/L, mean (SD)	1.1 (0.27)	1.1 (0.39)	1.2 (0.49)	2.0 (8.54)	1.3 (4.05)	0.266
Serum albumin, mmol/L, mean (SD)	36.9 (4.64)	37.0 (4.84)	37.0 (5.12)	36.3 (3.91)	36.8 (4.65)	0.538
Vitamin D, μg/L, mean (SD)	22.7 (11.39)	22.6 (11.79)	25.3 (11.56)	23.8 (11.81)	23.5 (11.65)	0.190
Bone mineral density						
BMD, *T*‐score, mean (SD)	−3.2 (0.84)	−3.0 (0.92)	−3.1 (1.06)	−2.9 (0.94)	−3.1 (0.94)	0.037
Outcomes						
Survival in index admission, *n* (%)	15 (9.0)	22 (15.1)	22 (17.2)	24 (18.3)	83 (14.5)	0.356
Length of stay, days, median (IQR)	8.6 (5.8)	8.7 (6.2)	9.7 (6.8)	10.1 (6.8)	9.1 (6.2)	0.002*

*Note*: Data presented as mean (SD) or median (IQR), whichever appropriate and frequency (percentage) for continuous and categorical data respectively. *p*‐values represent difference in characteristics based on ANOVA for continuous variables, and Chi‐square test for categorical variables.

Abbreviations: BMI, body mass index; BMD, bone mineral density; CKD, chronic kidney disease; eGFR, estimated glomerular filtration rate: HbA1c, glycated hemoglobin; IQR, Interquartile range; SD, standard deviation.

* *p* < 0.050.

### 3.2 Bone Health

The mean *T*‐score at the femoral neck was −3.1 ± 0.94. Notably, Group 2 (CKD−/DM+) exhibited a higher *T*‐score compared to Group 1 (CKD−/DM−), with an increase of 0.20 in BMD, as shown in Table 1. Group 4 (CKD+/DM+) similarly demonstrated a better *T*‐score than Group 1 (CKD−/DM−) (*p =* 0.04), as shown in Table 2.

**TABLE 2:** Differences in *T*‐score between patients with CKD and DM status.

Comparisons	Mean difference (95% CI)	*p* ^¥^
CKD−/DM+ vs. CKD−/DM−	0.250 (−0.070, 0.570)	0.101
CKD−/DM+ vs. CKD−/DM−	−0.150 (−0.480, 0.180)	0.533
CKD+/DM+ vs. CKD−/DM−	0.290 (0.030, 0.550)	0.042

*Note*: These are raw *p* values—not accounted for multiple comparisons. *p*¥ ‐ raw *p* value.

Abbreviations: BMD, bone mineral density; CI, confidence interval; CKD, chronic kidney disease; DM, diabetes mellitus.

### 3.3 Relationship of HbA1c to *T*‐Score

**FIGURE 1:**
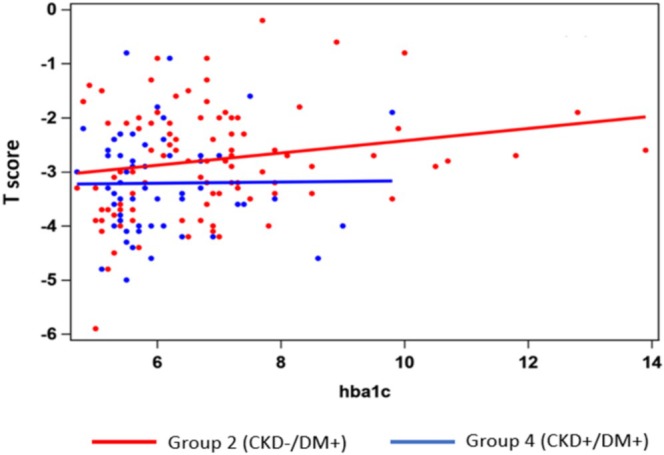
Comparison of *T* scores with worsening HBA1c in Group 2 versus Group 4.

Figure 1 illustrates the association between HbA1c and *T*‐score in Groups 2 and 4. Although a positive linear relationship was observed between higher HbA1c levels and *T*‐score, this relationship did not reach statistical significance. Notably, this positive association was absent in Group 4 (CKD+/DM+).

### 3.4 Clinical Outcomes

Patients in Group 4 had a longer length of stay compared to the other groups, with a mean of 10.1 days (*p* = 0.002); however, there was no significant difference in survival during the index admission between the groups.

## 4 Discussion

In this retrospective study of 571 elderly patients with fragility hip fractures, we examined the impact of CKD and DM on BMD in elderly patients with fragility hip fractures. Specifically, we explored the effects of CKD and DM when occurring alone or in combination, as well as the influence of worsening glycemic control, as measured by HbA1c, on BMD.

Our findings revealed that patients with DM alone exhibited a slightly higher *T*‐score (0.20) compared to those without DM or CKD. Interestingly, when DM and CKD coexisted, there was no detrimental effect on BMD, as assessed by *T*‐scores. However, in patients with T2DM, we observed a positive association between worsening HbA1c levels and *T*‐score. Notably, this association disappeared in the presence of concurrent CKD. Given the important role of serum phosphate and 25(OH) vitamin D levels in bone mineralization and formation, we have included this data alongside the *T*‐scores in Table 1. An inverse relationship between serum phosphate and BMD is well documented in both aging men and postmenopausal women [13, 14]. Hyperphosphatemia in CKD is known to contribute to bone fragility [15]. Similarly, vitamin D deficiency is often associated with or can exacerbate osteoporosis [16].

It is important to acknowledge that bone health is adversely affected by the presence of DM and CKD; yet conventional risk assessment tools often overlook these conditions [17, 18]. Our study adds to the existing literature by highlighting the differential effects of type 1 and type 2 diabetes on BMD, with type 2 diabetes being associated with higher BMD. This aligns with previous research indicating an increased BMD in patients with abnormal glucose metabolism [19, 20].

However, despite the apparent increase in BMD, individuals with diabetes are at a substantially higher risk (40%–70%) of fragility fractures compared to non‐diabetic counterparts [21, 22]. This increased fracture risk in type 2 diabetes is attributed to altered bone metabolism, leading to compromised bone microarchitecture, as evidenced by thicker femoral cortices and narrower bones, which predispose to microcrack accumulation and cortical porosity [4, 23]. Diabetes could affect bone through several mechanisms, some of which may have contradictory effects. Obesity, which is widespread in T2DM, is strongly associated with higher BMD, probably through mechanical loading and hormonal factors, including insulin, estrogen, and leptin [24, 25]. High‐resolution peripheral quantitative computed tomography (HRpQCT) has demonstrated a 10% higher trabecular BMD with an increase in intracortical porosity. An increased fraction of fat has also been reported in the bone marrow of patients with diabetes. Thus, these patients showing increased cortical porosity have significantly lower bone strength than healthy controls [26].

CKD, characterized by a markedly elevated fracture risk, is also known to negatively impact bone health through various mechanisms, including increased parathyroid hormone levels, reduced vitamin D levels, metabolic acidosis, and malnutrition. Unfortunately, established risk assessment tools like FRAX do not currently account for DM or CKD, which have significant implications for bone health [18].

Furthermore, therapeutic options for osteoporosis prevention in advanced CKD remain limited, highlighting an unmet need in this population [27]. Despite the expected detrimental effects of DM and CKD on BMD, our study did not find a compounding negative impact when these conditions coexisted.

There are various proposed mechanisms for increased BMD in patients with DM, one being the association with the deposition of glycosylated protein [21]. We found a positive correlation between higher HBA1C and *T*‐score; though it did not reach statistical significance. These findings are in line with a study from China reporting on the positive association of BMD with HBA1C when values exceeded 7.5% [28]. This association, however, was absent in the concurrent presence of CKD.

It is worth mentioning that our study has several limitations, including its cross‐sectional design, which precludes causal inference, and its restriction to elderly patients with fragility hip fractures. Our study did not include measures like trabecular bone score (TBS) or assessment by HR‐pQCT, which can predict hip and major osteoporotic fracture at least partly independent of BMD [29]. We also did not address the issue of falls, which is an important contributor to fracture risk in patients with T2DM. Our cohort of patients with CKD had an eGFR that was mildly decreased; hence, it would not be possible to comment on the effects of CKD on BMD in those with severe CKD or dialysis dependence. However, our findings underscore the importance of considering T2DM and CKD in the interpretation of BMD and clinical decision‐making regarding osteoporotic therapy initiation.

The interplay between T2DM and CKD on BMD underscores the complexity of bone health in these populations and highlights the need for comprehensive risk assessment strategies and targeted interventions to mitigate fracture risk in vulnerable individuals.

## 5 Conclusion

Our study among elderly patients who underwent hip fracture surgery revealed that Group 2 (CKD−/DM+) exhibited a significantly higher BMD compared to Group 1 (CKD−/DM−), with an increase of 0.20 in *T*‐score. This underscores the potential influence of DM on bone health in this population.

Contrary to expectations, the presence of both CKD and DM did not contribute to a worsened BMD. This suggests that the effect of T2DM on the BMD was overwhelming, at least in a cohort of patients with mild CKD.

Our findings indicate a positive association between higher HbA1c levels and BMD in Group 2 (CKD−/DM+), suggesting a potential protective effect of elevated HbA1c on bone health. However, this correlation is not particularly robust, indicating that other factors may also influence BMD in this group. Interestingly, this positive association disappears in Group 4 (CKD+/DM+), suggesting a different mechanism affecting bone health in individuals with both CKD and DM.

Furthermore, we observed a significant difference in postoperative length of stay (LOS) between Group 1 (CKD−/DM−) and Group 4 (CKD+/DM+), with Group 4 experiencing a longer LOS (median 10.1 days vs. 8.6 days, *p* = 0.002). This highlights the potential impact of CKD and DM comorbidity on postoperative recovery and hospitalization duration in elderly hip fracture patients.

### 5.1 Implications

These findings underscore the importance of early detection and management of DM and CKD in elderly patients to mitigate the risk of fragility hip fractures and improve bone health. Future research should delve deeper into the mechanisms underlying the observed associations and explore targeted interventions to optimize bone health outcomes in this vulnerable population.

## Author Contributions

Debajyoti Roy conceived the study. Rehana Sultana conducted data analysis. Debajyoti Roy, Jiashen Cai, Chee Yong Ng, Sreekanth Koduri, Chang Yin Chionh, Rehana Sultana, and Wenxiang Yeon discussed the results and contributed to the final manuscript.

## Ethics Statement

The study received ethical approval from the Singhealth Institutional Review Board (Approval No: 2017/2962), with informed consent waived by the board.

## Conflicts of Interest

The authors declare no conflicts of interest.

## Data Availability

The datasets used in this study are available from the corresponding author, Dr. Debajyoti Roy, upon reasonable request.

## References

[1] B. Prasad , T. Ferguson , N. Tangri , C. Y. Ng , and T. L. Nickolas , “Association of Bone Mineral Density With Fractures Across the Spectrum of Chronic Kidney Disease: The Regina CKD‐MBD Study,” Canadian Journal of Kidney Health and Disease 6 (2019): 205435811987053, 10.1177/2054358119870539. PMC6704416 31467681

[2] P. Vestergaard , “Discrepancies in Bone Mineral Density and Fracture Risk in Patients With Type 1 and Type 2 Diabetes—A Meta‐Analysis,” Osteoporosis International 18 (2007): 427–444, 10.1007/s00198-006-0253-4. 17068657

[3] M. Jang , H. Kim , S. Lea , S. Oh , J. S. Kim , and B. Oh , “Effect of Duration of Diabetes on Bone Mineral Density: A Population Study on East Asian Males,” BMC Endocrine Disorders 18 (2018): 61, 10.1186/s12902-018-0290-y. 30185190 PMC6126021

[4] L. Oei , M. C. Zillikens , A. Dehghan , et al., “High Bone Mineral Density and Fracture Risk in Type 2 Diabetes as Skeletal Complications of Inadequate Glucose Control: The Rotterdam Study,” Diabetes Care 36 (2013): 1619–1628, 10.2337/dc12-1188. 23315602 PMC3661786

[5] S. Joad , E. Ballato , F. Deepika , et al., “Hemoglobin A1c Threshold for Reduction in Bone Turnover in Men With Type 2 Diabetes Mellitus,” Frontiers in Endocrinology 12 (2021): 788107. 35027909 10.3389/fendo.2021.788107 PMC8750620

[6] A. V. Schwartz , J.‐Y. C. Backlund , I. H. de Boer , et al., “Risk Factors for Lower Bone Mineral Density in Older Adults With Type 1 Diabetes: A Cross‐Sectional Study,” Lancet Diabetes and Endocrinology 10 (2022): 509–518, 10.1016/S2213-8587(22)00103-6. 35576955

[7] D. Roy , S. Pande , S. Thalanki , et al., “Hip Fractures in Elderly Patients With Non‐Dialysis Dependent Chronic Kidney Disease: Outcomes in a Southeast Asian Population,” Medicine (Baltimore) 100 (2021): e26625, 10.1097/MD.0000000000026625. 34232221 PMC8270610

[8] A. S. Levey , L. A. Stevens , C. H. Schmid , et al., “A New Equation to Estimate Glomerular Filtration Rate,” Annals of Internal Medicine 150 (2009): 604–612, 10.7326/0003-4819-150-9-200905050-00006. 19414839 PMC2763564

[9] A. Levin and P. E. Stevens , “Summary of KDIGO 2012 CKD Guideline: Behind the Scenes, Need for Guidance, and a Framework for Moving Forward,” Kidney International 85 (2014): 49–61, 10.1038/ki.2013.444. 24284513

[10] K. H. Leong and P. H. Feng , “Bone Mineral Density Measurements Using the Hologic QD2000 in 175 Singaporean Women Aged 20–80,” Singapore Medical Journal 38, no. 1 (1997): 25–26. 9269350

[11] F. Thoo , S. Chng , K. Lam , et al., “To Establish the Normal Bone Mineral Density Reference Database for the Singapore Male,” Annals of the Academy of Medicine, Singapore 31 (2002): 21–25. 11885490

[12] J. A. Kanis , “Assessment of Fracture Risk and Its Application to Screening for Postmenopausal Osteoporosis: Synopsis of a WHO Report,” Osteoporosis International 4 (1994): 368–381, 10.1007/BF01622200. 7696835

[13] E. O. Billington , G. D. Gamble , S. Bristow , and I. R. Reid , “Serum Phosphate Is Related to Adiposity in Healthy Adults,” European Journal of Clinical Investigation 47 (2017): 486–493, 10.1111/eci.12769. 28517037

[14] B. L. Clarke , P. R. Ebeling , J. D. Jones , et al., “Predictors of Bone Mineral Density in Aging Healthy Men Varies by Skeletal Site,” Calcified Tissue International 70 (2002): 137–145, 10.1007/s00223-001-1072-4. 11907709

[15] A. Pimentel , P. Ureña‐Torres , M. C. Zillikens , J. Bover , and M. Cohen‐Solal , “Fractures in Patients With CKD—Diagnosis, Treatment, and Prevention: A Review by Members of the European Calcified Tissue Society and the European Renal Association of Nephrology Dialysis and Transplantation,” Kidney International 92 (2017): 1343–1355, 10.1016/j.kint.2017.07.021. 28964571

[16] P. Lips and N. M. van Schoor , “The Effect of Vitamin D on Bone and Osteoporosis,” Best Practice & Research. Clinical Endocrinology & Metabolism 25 (2011): 585–591, 10.1016/j.beem.2011.05.002. 21872800

[17] P. M. Camacho , S. M. Petak , N. Binkley , et al., “American Association of Clinical Endocrinologists/American College of Endocrinology Clinical Practice Guidelines for the Diagnosis and Treatment of Postmenopausal Osteoporosis‐2020 Update,” Endocrine Practice 26 (2020): 1–46, 10.4158/GL-2020-0524SUPPL. 32427503

[18] J. A. Kanis , E. V. McCloskey , H. Johansson , et al., “Development and Use of FRAX in Osteoporosis,” Osteoporosis International 21, no. S2 (2010): S407–S413, 10.1007/s00198-010-1253-y. 20464374

[19] A. Räkel , O. Sheehy , E. Rahme , and J. LeLorier , “Osteoporosis Among Patients With Type 1 and Type 2 Diabetes,” Diabetes & Metabolism 34 (2008): 193–205, 10.1016/j.diabet.2007.10.008. 18308607

[20] B. Liu , J. Liu , J. Pan , C. Zhao , Z. Wang , and Q. Zhang , “The Association of Diabetes Status and Bone Mineral Density Among US Adults: Evidence From NHANES 2005–2018,” BMC Endocrine Disorders 23 (2023): 27, 10.1186/s12902-023-01266-w. 36721144 PMC9890809

[21] A.‐K. Picke , G. Campbell , N. Napoli , L. C. Hofbauer , and M. Rauner , “Update on the Impact of Type 2 Diabetes Mellitus on Bone Metabolism and Material Properties,” Endocrine Connections 8 (2019): R55–R70, 10.1530/EC-18-0456. 30772871 PMC6391903

[22] S. Kurra , D. A. Fink , and E. S. Siris , “Osteoporosis‐Associated Fracture and Diabetes,” Endocrinology and Metabolism Clinics of North America 43 (2014): 233–243, 10.1016/j.ecl.2013.09.004. 24582100

[23] L. M. Giangregorio , W. D. Leslie , L. M. Lix , et al., “FRAX Underestimates Fracture Risk in Patients With Diabetes,” Journal of Bone and Mineral Research 27 (2012): 301–308, 10.1002/jbmr.556. 22052532

[24] M. Wakasugi , R. Wakao , M. Tawata , N. Gan , K. Koizumi , and T. Onaya , “Bone Mineral Density Measured by Dual Energy X‐Ray Absorptiometry in Patients With Non‐Insulin‐Dependent Diabetes Mellitus,” Bone 14 (1993): 29–33, 10.1016/8756-3282(93)90252-6. 8442999

[25] I. R. Reid , M. C. Evans , G. J. Cooper , R. W. Ames , and J. Stapleton , “Circulating Insulin Levels Are Related to Bone Density in Normal Postmenopausal Women,” American Journal of Physiology 265 (1993): E655–E659, 10.1152/ajpendo.1993.265.4.E655. 8238341

[26] J. N. Farr , M. T. Drake , S. Amin , L. J. Melton, III , L. K. McCready , and S. Khosla , “In Vivo Assessment of Bone Quality in Postmenopausal Women With Type 2 Diabetes,” Journal of Bone and Mineral Research 29 (2014): 787–795, 10.1002/jbmr.2106. 24123088 PMC3961509

[27] P. Evenepoel , J. Cunningham , S. Ferrari , et al., “Diagnosis and Management of Osteoporosis in Chronic Kidney Disease Stages 4 to 5D: A Call for a Shift From Nihilism to Pragmatism,” Osteoporosis International 32 (2021): 2397–2405, 10.1007/s00198-021-05975-7. 34129059

[28] L. Gao , Y. Liu , M. Li , et al., “Based on HbA1c Analysis: Bone Mineral Density and Osteoporosis Risk in Postmenopausal Female With T2DM,” Journal of Clinical Densitometry 27 (2023): 101442, 10.1016/j.jocd.2023.101442. 38039558

[29] E. Shevroja , J.‐Y. Reginster , O. Lamy , et al., “Update on the Clinical Use of Trabecular Bone Score (TBS) in the Management of Osteoporosis: Results of an Expert Group Meeting Organized by the European Society for Clinical and Economic Aspects of Osteoporosis, Osteoarthritis and Musculoskeletal Diseases (ESCEO), and the International Osteoporosis Foundation (IOF) Under the Auspices of WHO Collaborating Center for Epidemiology of Musculoskeletal Health and Aging,” Osteoporosis International 34 (2023): 1501–1529, 10.1007/s00198-023-06817-4. 37393412 PMC10427549

